# Development of an Explainable Machine Learning Computational Model for the Prediction of Severe Complications After Orchiectomy in Stallions

**DOI:** 10.3390/ani16030377

**Published:** 2026-01-25

**Authors:** Panagiota Tyrnenopoulou, Dimitris Kalatzis, Yiannis Kiouvrekis, Eugenia Flouraki, Leonidas Folias, Epameinondas Loukopoulos, Alexandros Starras, Panagiotis Chalvatzis, Vassiliki Tsioli, Vasia S. Mavrogianni, George C. Fthenakis

**Affiliations:** 1 Veterinary Faculty, University of Thessaly, 43100 Karditsa, Greece; eflouraki@uth.gr (E.F.); eloukopoulos@uth.gr (E.L.); vtsioli@uth.gr (V.T.); vmavrog@vet.uth.gr (V.S.M.); 2 Faculty of Public and One Health, University of Thessaly, 43100 Karditsa, Greece; dkalatzis@uth.gr (D.K.); kiouvrekis.y@uth.gr (Y.K.); 3 Business School, University of Nicosia, Nicosia 1700, Cyprus; 4 Department of Information Technologies, University of Limassol, Limassol 3020, Cyprus; 5 Private Veterinary Practice, 41110 Larissa, Greece; 6 Private Veterinary Practice, 73100 Chania, Greece; starrasvet@gmail.com; 7 Private Veterinary Practice, 50100 Kozani, Greece; chalvatzispanagiotisvet@hotmail.com

**Keywords:** artificial intelligence, castration, Gradient Boosting, horse, Logistic Regression, orchiectomy, post-surgery complication, prediction, Random Forest, testis

## Abstract

The objective of the present study was to apply supervised Machine Learning to predict severe complications after equine orchiectomy. A dataset of 612 cases of orchiectomies in stallions was used for the development of a computational model, among which in 8.5% of cases severe complications (colic, continued stallion-like behaviour, evisceration, funiculitis, haemorrhage, and scrotal infection) were diagnosed post-orchiectomy. Three supervised Machine Learning tools were employed: Logistic Regression (12 different models evaluated), Random Forest (64 models), and Gradient Boosting (8 models). For the prediction of the development of severe complications post-orchiectomy, Logistic Regression was the tool that produced the best discrimination measures, where accuracy, precision and recall were 0.9134, 0.8391, and 0.9133, respectively. The results of the analysis for SHapley Additive exPlanations values for the impact of the independent variables in the prediction of the development of complications indicated that (a) the age of the horse and (b) the surgical technique employed were the two variables that mostly influenced the prediction outcome, findings that were unambiguous in the models developed by any Machine Learning tool. The findings of this study indicate that computational models could be used as adjunct tools to support clinical decisions in the peri-operative management of horses.

## Simple Summary

The present work describes the development of a model, through the use of Machine Learning methodologies, for making predictions regarding the possible development of complications after an orchiectomy (castration) in stallions. The study has been based on a large dataset of 612 cases of surgery in stallions, operated in conditions of general veterinary practice by one of three experienced veterinary surgeons. Supervised Machine Learning methodologies were applied and, in total, 84 models were evaluated. The recall rate of the selected models was over 90%. The findings of this study have indicated that computational models could be used as adjunct tools to support clinical decisions in the peri-operative management of horses.

## 1. Introduction

In stallions, an orchiectomy is a frequently performed surgical procedure by veterinarians active in equine practice. The operation is indicated for the management (reduction) of undesirable (aggressive) behaviour of stallions, for the prevention of reproductive activity (undesired breeding) by male horses, and for the surgical treatment of reproductive disorders (e.g., inguinal herniation, testicular neoplasia) [1,2]. An orchiectomy is considered a routine procedure, but it can be followed by a wide array of complications, nevertheless. These may range from mild scrotal oedema to severe, potentially life-threatening events, e.g., haemorrhage, evisceration, or septic funiculitis [3,4]. Although severe complications after an orchiectomy are uncommon in stallions, they pose major concerns in equine surgery [2]. The severe complications of orchiectomies adversely affect the welfare of the operated animals [3,4], and can also have financial consequences for the animal owners [5]. The development and the severity of these complications may be influenced by several factors [2,6].

Traditional risk analysis methods typically based on Logistic Regression can capture linear associations between a few predefined variables, but it may fail to identify non-linear interactions or subtle patterns hidden within multidimensional datasets [7]. This limitation underscores the need for advanced analytical techniques capable of recognising complex interdependencies within data and providing more reliable predictive capability.

Machine Learning is a pivotal subfield of Artificial Intelligence dedicated to the design and implementation of algorithmic models that possess the intrinsic ability to automatically learn intricate patterns and relationships from empirical data, thereby continually optimising their performance and delivering high-fidelity predictions without the necessity of explicit, static programming [8]. In supervised Machine Learning applications, algorithms are trained to recognise patterns and to make predictions or classifications based on input features. Machine Learning methodologies offer important benefits in providing predictions, which can be achieved by detecting complex patterns within large datasets. That way, automation, higher accuracy, and quick and easy adaptability can be achieved in comparison to traditional methods. Within the present framework, such approaches will allow veterinarians to make data-driven decisions, objectively and with reduced human bias, with the result to individualise surgical work and operative actions, ultimately improving animal welfare [9].

Recent advances have shown the applicability of Machine Learning approaches in animal health. For example, Kiouvrekis et al. [10] applied both supervised and unsupervised Machine Learning algorithms to predict the prevalence of subclinical mastitis in dairy sheep farms, achieving high classification accuracy and demonstrating that such models can identify complex, multifactorial interactions within field data. Such findings exemplify how Machine Learning approaches are now being adopted across veterinary medicine to address diagnostic and prognostic challenges in a range of clinical contexts [11]. Published studies have demonstrated the utility of Machine Learning for predicting surgical outcomes, classifying disease severity and improving diagnostic efficiency in equine colic, lameness assessments, and metabolic disorders [11,12,13]. A recent bibliometric study for veterinary applications of artificial intelligence (in which a large array of search terms, including ‘machine learning’, ‘artificial intelligence’, ‘decision tree’, ‘support vector machine’, and ‘neural network’, was evaluated) revealed that the majority of relevant studies focused on diagnoses of disease and prediction of treatment outcomes [14]. However, no work has yet been reported about the use of Machine Learning for predicting severe complications following orchiectomies of stallions. Given the frequency of performing this procedure and the clinical impact of its complications, this represents a significant gap in the literature.

In view of the above, there is a scope to develop a Machine Learning model that could be used to predict the potential development of severe complications post-orchiectomy in stallions. The specific objective of the present study was to apply supervised Machine Learning to predict severe complications after equine orchiectomy. The study involved the analysis of data from cases of orchiectomy performed in field conditions and the application of Machine Learning tools in order to enhance identification of high-risk cases and provide evidence-based peri-operative management of horses.

## 2. Materials and Methods

### 2.1. Animal Data and Dataset Used for the Construction of the Computational Model

The dataset included 612 cases of orchiectomy of stallions, which had been performed in Greece. The clinical records of stallions, in which orchiectomies were performed between September 2022 and March 2024 in Greece, by one of three different veterinary surgeons (all with over five years of relevant clinical experience), were taken into account.

Standard pre-operative procedures were carried out on all horses starting six weeks before the planned surgery (Table S1). For each horse, the following information regarding characteristics of the horses were obtained from the clinical records: age, breed (recorded by the clinical veterinarian during the pre-operative examination of the animal), and bodyweight (also recorded by the clinical veterinarian by using a measuring tape (Elico Equine Height and Weight Measuring Tape, Equistore, Rathfarnham, Co., Dublin, Ireland)). Analgesic–anaesthetic procedures performed differed in accordance with the performance of the operation with the animal standing or in recumbency, and included pre-medication, anaesthesia (specifically for animals operated in recumbency), and local analgesia (Table S2).

Orchiectomies were performed using one of three principal surgical techniques: open, semi-closed, or closed technique (Table S3). The surgical approach to the testes was performed through a scrotal incision. In general, the surgical part of the work was performed as detailed by Kilcoyne and Spier [2], Rodden et al. [4], Baldwin [6], Moll et al. [15], Carmalt et al. [16], Schumacher [17], and Kilcoyne et al. [18] (Table S4). At the end of the surgical procedure, haemostasis was achieved by means of one of various procedures, including the use of the Henderson instrument, the use of the Reimer emasculator, the ligation of the testicular artery, or combinations thereof [3,6,17,18,19,20] (Table S4).

Animals were post-operatively monitored by their owners in accordance with instructions given by the clinical veterinarians. Post-operative management also included restriction of animals within their boxes initially with mild walking thereafter, cold hosing of the genital area, pharmaceutical treatment (administration of flunixin meglumine and antibiotics (penicillin and streptomycin (Penicillin Streptomycin 200/200^®^; MSD Animal Health, Rathway, NJ, USA)), and avoidance of mixing with mares. The development of complications was promptly reported by the owners. Moreover, animals were routinely re-examined by the surgeons who operated on them on the day after the operation, as well as 9 to 12 days later. In cases of observed complications, which were reported by the owners, a visit was performed by the surgeon who operated on the horse within 12 h. In such cases, a detailed clinical examination was performed. The surgical complication(s) were verified, identified, and then recorded appropriately.

The details regarding the operation (the position of animal during the operation, the surgical technique employed, and the haemostasis procedure applied), as well as the development of the complications after the surgery, were also recorded by the clinical veterinarians on standard clinical records. These were maintained by the clinical veterinarians as part of equine veterinary work performed professionally.

The variables used for the construction of the computational models are described in Table 1.

Throughout the procedure for the development of the computational model, we performed the following general steps: (a) we defined the problem, (b) we established the desired outcome, (c) we prepared the data, (d) we carried out feature scaling, (e) we carried out splitting of the data and then evaluated the model, and (f) we tuned the hyperparameters. These multiple considerations ultimately ensured the efficacy, robustness, and applicability of the computational model that had been developed.

### 2.2. Construction of the Computational Model

During preprocessing, the raw variables were prepared in a way that Machine Learning algorithms received stable, comparable inputs, and the same transformations were applied consistently at training and inference time. Numeric variables were standardised using StandardScaler, with the aim to centre them and scale to unit variance; this action stabilised optimisation for linear models and prevented features with larger magnitudes from dominating the objective. Categorical variables were treated as nominal and expanded through one-hot encoding. All steps were composed with sklearn’s ColumnTransformer and Pipeline, ensuring that the exact same preprocessing was bound to the model and automatically executed during both fit and predict calls. This design improved reproducibility, guarded against leakage, and simplified deployment as the pipeline serialised as a single artefact.

Three Machine Learning tools were employed with the aim to establish a strong baseline and to capture both linear and non-linear relationships in the dataset. These tools were: (a) Logistic Regression, (b) Random Forest, and (c) Gradient Boosting. The hyperparameters employed for each of the above models are presented in Table 2. In total, 84 models were evaluated during this stage of the assessment. Model development followed an 80/20 stratified train/test split which preserved the original class ratio in both folds. Hyperparameters were selected using cross-validation on the training fold in order to avoid overfitting the test set.

### 2.3. Data Management and Analysis

For the selection of the best model, the following discrimination measures were used: accuracy, precision, recall, and F1 score. Additionally, ROC AUC (Receiver operating characteristic Area under the curve) and PR AUC (Precision recall Area under the curve) were also used, because the minority class in the dataset was at a low proportion (8.5%), hence, test-set accuracy on its own might have been misleading.

Within each Machine Learning tool, the model with the best discrimination metrics was selected. Subsequently, these three selected models (i.e., one among all models within each tool) were compared between them. The Kruskal–Wallis test was employed for these comparisons. Statistical significance was set at *p* < 0.05.

Finally, analysis of the importance of the independent variables used in predicting the development of severe complications after an orchiectomy was performed using SHapley Additive exPlanations (SHAP) values analysis through the use of the SHAP Python library (version 0.49.1).

## 3. Results

### 3.1. Details of Stallions Operated for Orchiectomy and Post-Operative Complications Observed

The median age of the horses, which was recorded based on the identification document of each animal, was 9 years (interquartile range (IQR): 9 years). The median bodyweight of the animals, which was recorded by using a measuring tape (Elico Equine Height and Weight Measuring Tape, Equistore, Rathfarnham, Co., Dublin, Republic of Ireland), was 380 kg (IQR: 230 kg). In total, horses comprised 11 different breeds, most frequently mixed-breed (30.7% of all animals) or Shetland ponies (18.3%) (Table S5).

Each of the three surgeons who had performed orchiectomies contributed with a different number of cases in the study (*n* = 364, *n* = 81, and *n* = 187), in accordance with their respective clinical loads.

Severe post-operative complications were observed in 52 horses (8.5% (95% confidence interval (CI): 6.5–11.0%)). These included the following: colic, continued stallion-like behaviour, evisceration, funiculitis, haemorrhage, and scrotal infection (Table 3). Notably, all these complications are considered to require intense veterinary intervention and care, and may occasionally even become life-threatening. There was no significant difference between the three surgeons in the proportion of horses operated on by each of them which subsequently developed severe complications (*p* = 0.07). Ultimately, among the horses with severe complications, one was euthanized (1.9% (95% CI: 0.3–10.1%)), based on welfare grounds, as it was deemed to be beyond treatment, whilst all the other animals (*n* = 51) recovered fully.

**Figure 1:**
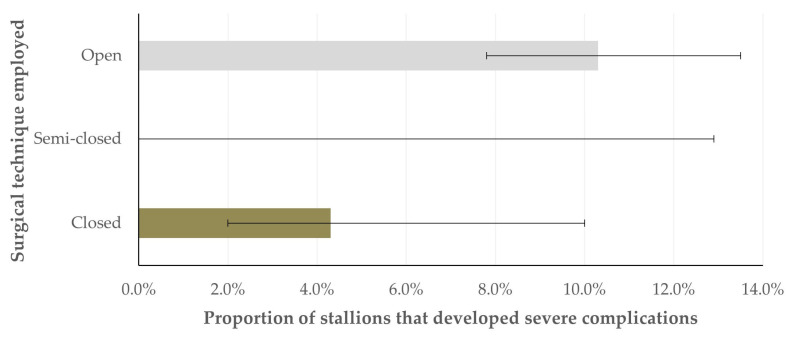
Bar plot of the proportion of stallions that developed severe complications after an orchiectomy in accordance with the surgical technique employed in the operation (bars indicate 95% confidence intervals).

The development of severe complications was more frequent in stallions in which an open surgical technique was employed (10.3% of horses in which the technique was employed (95% CI: 7.8–13.5%)). In contrast, horses operated on using a closed or a semi-closed technique developed complications significantly less often: 4.3% (95% CI: 2.0–10.0%) and 0.0% (95% CI: 0.0–12.9%) (*p* = 0.022) (Figure 1). The median age of horses which developed severe complications post-operatively was greater than the age of horses with no complications: 11 (interquartile age (IQR): 8; minimum–maximum: 2–19) years versus 9 (IQR: 9; min.–max.: 2–19) years (*p* = 0.05).

No significant associations emerged between any the six severe complications observed with the surgical technique employed (*p* > 0.06 for all evaluations) and the age of the animals (*p* > 0.12 for all evaluations).

### 3.2. Selection of Computational Model

**Figure 2:**
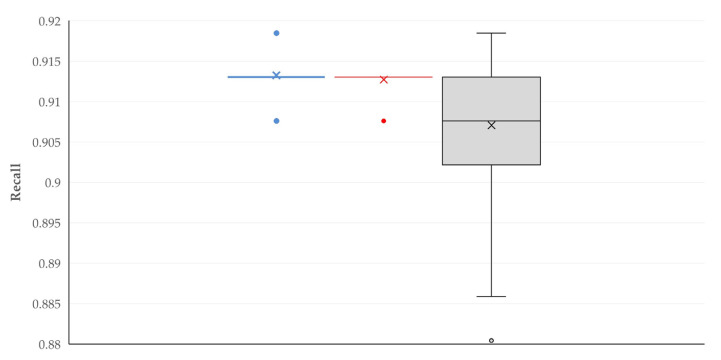
Box-and-whisker plot of recall for the model with the best discrimination metrics within each Machine Learning tool employed for the prediction of severe complications after orchiectomies in stallions (blue plot: Logistic Regression, red plot: Random Forest, and grey plot: Gradient Boosting).

**Figure 3:**
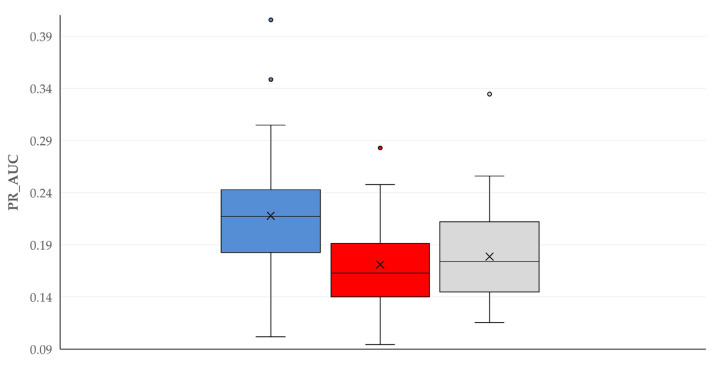
Box-and-whisker plot of PR AUC (Precision recall Area under the curve) for the model with the best discrimination metrics within each Machine Learning tool employed for the prediction of severe complications after orchiectomies in stallions (blue plot: Logistic Regression, red plot: Random Forest, and grey plot: Gradient Boosting).

A summary of the performance of the model with the best discrimination metrics within each Machine Learning tool is in Table 4. The comparison of these three models revealed a significant difference between them for accuracy (*p* < 0.0001 between models) (Figure S1), recall (*p* < 0.0001) (Figure 2), F1 score (*p* = 0.033) (Figure S2), and PR AUC (*p* < 0.0001) (Figure 3), in which Logistic Regression showed the best performance. No significant differences between the models were seen for precision (*p* = 0.19) and ROC AUC (*p* = 0.29).

Based on these results, Logistic Regression was considered as the tool with the best performance for the prediction of severe complications after orchiectomies in stallions.

### 3.3. Analysis of SHAP Values

**Figure 4:**
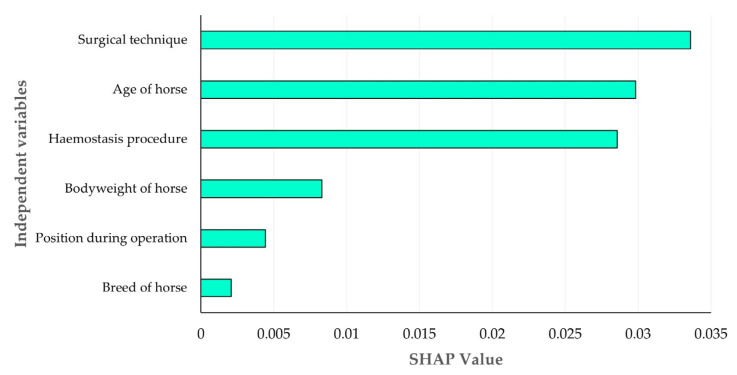
Bar plot of mean absolute SHapley Additive exPlanations values for the importance of six independent variables employed in the prediction of development of severe complications after orchiectomies in stallions through the use of Logistic Regression.

**Figure 5:**
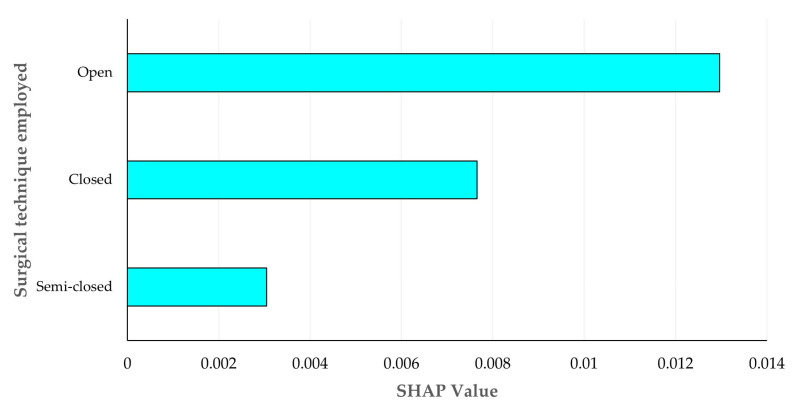
Bar plot of mean absolute SHapley Additive exPlanations values for the importance of three surgical techniques employed in the prediction of development of severe complications after orchiectomies in stallions through the use Logistic Regression.

The results of the analysis for SHAP values for the impact of the six independent variables in the prediction of the development of severe complications after an orchiectomy indicated that (a) the age of the horse and (b) the surgical technique employed were the two variables that mostly influenced the prediction outcome (Figure 4). The major importance of these two variables was unambiguous in the models developed by any of the three Machine Learning tools (Table S6). The median age of the horses that developed complications was significantly higher than the age of the horses which did not: 11 years (interquartile range: 4) versus 9 years (interquartile range 4.5) (Figure S3). Among the categories of surgical techniques employed, the open technique was the one that mostly influenced the prediction outcome (Figure 5). The major importance of this category was again unambiguous in the models developed by any of the three Machine Learning tools (Table S7).

## 4. Discussion

The study has provided a computational model for the prediction of the development of severe complications after orchiectomies in stallions, which is a surgical procedure performed frequently in equine veterinary work. The development of such tools will be of benefit to veterinarians active in equine practice, who can thus have reliable information regarding adverse outcomes of the operation.

### 4.1. Machine Learning Methodology

#### 4.1.1. Machine Learning Tools Employed in the Development of the Model

In the present study, three Machine Learning tools were employed to develop the best computational model. Logistic Regression provided a transparent, regularised linear baseline, in which calibrated probabilities and standardised coefficients aided interpretability; with class weighting, the model was often competitive under imbalance [21]. Random Forest introduced non-linear decision boundaries and robust feature interactions via an ensemble of decorrelated decision trees, typically offering strong performance with minimal tuning [22]. Gradient Boosting (sklearn) incrementally built an additive ensemble of shallow trees to reduce bias, trading off learning rate and number of estimators to capture subtler patterns while controlling overfitting [23,24,25,26].

#### 4.1.2. Discrimination Measures Employed in the Evaluation of the Model

An important methodological consideration was the class imbalance, as positive cases (i.e., horses that developed complications) accounted for 8.5% of all data. In such settings, the use of accuracy alone as a discrimination metric may be misleading, because a high accuracy may be achieved by models that predominantly predicted the majority class. In order to address this issue, several complementary strategies were adopted: first, stratified train-test splitting was applied to preserve the original class distribution across data partitions; and second, model performance was evaluated using an array of discrimination measures that could be more informative in cases of class imbalance, including precision, recall, F1 score, and, importantly, PR AUC, which directly reflected performance on the minority class. Synthetic oversampling techniques, such as the Synthetic Minority Over-sampling Technique (SMOTE), were not applied in the present analysis. Although such approaches could increase minority class recall, they would generate artificial samples that might not fully represent the biological and clinical variability inherent in real-world surgical cases. This would be particularly relevant in heterogeneous field datasets, in which synthetic instances might introduce unrealistic feature combinations or amplify noise. Given the moderate level of imbalance and the clinical significance for the preservation of authentic case distributions, the present study favoured evaluation metrics, stratification, and class weighting over data-level resampling. This strategy aimed to balance predictive performance with clinical plausibility and model generalisability.

Accuracy represents the proportion of correct predictions among all the predictions made by the model; the measure is intuitive, but may be misleading if most animals in a study were healthy, which is particularly true in cases of class imbalance (referring to one class including significantly more samples than other classes, causing models to favour the majority class and perform poorly on the minority class(es)). Precision measures the proportion of positive predictions that are truly positive, hence high precision indicates a low false-positive rate. Recall measures the proportion of actual positive cases that are correctly identified; thus, high recall will reflect a reduction in false negatives. The F1 score is the harmonic mean of precision and recall, balancing both and providing a single measure that can balance the trade-off between those two, which is helpful when a compromise between catching every case and avoiding false positives is needed; the measure is particularly useful when dealing with imbalanced datasets. ROC AUC analysis is the gold-standard method for evaluating how effectively a binary classification model can distinguish between two clinical outcomes; in such cases, the ROC curve provides a visual representation of the trade-off between sensitivity (the true positive rate) and [1—specificity] (i.e., the false positive rate) across the full spectrum of possible diagnostic thresholds. Therein, the Area under the curve (AUC) serves as a single, threshold-independent summary of a model’s discriminative power; in clinical settings, the ROC AUC represents the probability that the model will assign a higher risk score to a randomly selected animal who developed a severe complication compared to a randomly selected animal who did not. PR AUC summarises the trade-off between precision and recall across different decision thresholds and measures the model performance across different thresholds; it is considered to be superior to ROC AUC when dealing with infrequent clinical outcomes as it focuses specifically on the ‘positive’ class within a dataset [27,28].

From a clinical standpoint, recall may likely be the most critical measure in this application, because failure to identify a horse at risk of severe complications could result in insufficient monitoring or delayed intervention. The relatively high recall achieved by the Logistic Regression model (>91%) suggested that this might be suitable as screening or decision-support tools. Precision, while lower in the models developed by these two tools, remained acceptable in the context of clinical decision-making, as false positives may lead to increased vigilance rather than harmful interventions. Finally, the use of PR AUC further supported the robustness of the selected model under class imbalance and also placed Logistic Regression as the most appropriate tool and model for use in the problem being studied.

#### 4.1.3. Use of SHapley Additive exPlanations Employed in the Interpretation of the Model

Possible limitations of the developed model include reduced transparency in model decisions and problems with overfitting in the model. For the former, the use of SHAP, an explainable technique, has provided a degree of influence in each one of the independent variables used in building the computational model, and thus has helped to interpret and ascertain the importance of these variables. For the latter, the application of 50 evaluations within the given dataset, as part of the procedure, using resampling, shuffling, and the k-fold cross-validation method and the implementation of cross-validation to assess the performance of the model, contributed to minimising potential problems. All of the above has contributed to breaking down the complex algorithmic decisions into comprehensible components [29].

SHapley Additive exPlanations (SHAP) values analysis is a means to explain the output of a computational model based on Machine Learning methodology. The technique was employed in order to understand how the individual variables used in the computational model influenced the predictions when using the model [30]. SHAP quantified feature importance based on principles of game theory and revealed how each feature contributed to the final output of the model. Through the use of the SHAP Python library, SHAP values were calculated for each prediction [30]. SHAP values can provide a model-agnostic measure of feature importance based on each variable’s contribution to individual predictions. In contrast to odds ratios, which describe average population-level effects, SHAP values can capture how strongly a variable influences model behaviour across all observations. Mean absolute SHAP values can quantify the relative importance of predictors, but do not indicate the direction of their effect. SHAP values are grounded in cooperative game theory and satisfy properties such as fairness and consistency. Therefore, in the present work, the results of this analysis reflect how strongly each independent variable influences model predictions rather than whether that variable would increase or decrease the probability of severe complications after an orchiectomy.

### 4.2. Influence of Independent Values in the Models Constructed

The severe complications studied in the present work require immediate and intense veterinary care, and may occasionally even become life-threatening. In any case, the two independent variables which appeared to have the most influence in the models were the same regardless of the tool employed. Older horses were associated with a higher risk for complications than younger; this may occur as the result of increased number of matings compared to younger horses, as well as additional animal characteristics, e.g., increased testicular size, vascular diameter, and connective tissue density, which are established predisposing factors for haemorrhages or delayed healing of wounds [6], findings which confirmed an earlier report regarding the identification of older age as a risk factor for development of post-orchiectomy complications [31]. The association of the open surgical orchiectomy technique with a higher incidence of complications post-operatively can be the consequence of the exposure of the vaginal tunic of the animals, an approach that may facilitate and contribute to infection of the surgical area [2]. In general, open orchiectomy techniques, particularly those involving field conditions and second intention healing, are widely recognised as being associated with a higher incidence of post-operative complications [6].

The computational model developed in this study can serve as a decision-support tool in clinical equine veterinary practice. Under field conditions, model-derived risk estimates could be used to stratify cases in accordance with their likelihood for development of post-operative complications. The model can be used to support decisions regarding potential referral of horses for orchiectomies to a veterinary hospital, as well as the selection of surgical methodology or the intensity of post-operative management. In this context, the model is intended to support risk-informed planning and communication.

Specifically, with regard to post-operative management, it is noted that under field conditions some variability in the conditions of animal care is expected. This arises from housing conditions and the general environment of the animal, as well as the lack of adherence to the prescribed instructions from the part of the owner. Therefore, it can contribute to a differentiation in the predicted outcome of the surgery, because expected standards of post-operative care would not be met.

## 5. Conclusions

The findings of this study indicate that Machine Learning algorithms can be usefully employed for the prediction of the potential development of severe complications after orchiectomy in stallions. These computational prediction models were developed using data collected during clinical veterinary work. The models showed very good performance and thus can be employed as adjunct tools to support clinical decisions in the peri-operative management of horses. The model developed can serve as a decision-support tool in clinical equine veterinary practice.

## Figures and Tables

**Table 1:** Variables used for the construction of the computational model.

**1. Target Value**
Development of severe complications following an orchiectomy in a stallion (binary)
**2. Independent Variables**
Age of horse (numeric)
Bodyweight of horse (numeric)
Breed of horse (categorical)
Position of horse during the operation (binary)
Surgical technique employed (categorical)
Haemostasis procedure applied (categorical)

**Table 2:** Supervised Machine Learning tools employed, hyperparameters used for each tool, and numbers of models produced during the assessment of the study problem.

Machine Learning Tool	No. of Different Models Evaluated	Hyperparameter	Domain/Values
Logistic Regression	12	solver	*lbfgs*, *liblinear*
		penalty	*l*2
		C	0.1, 1.0, 5.0
		class_weight	none, ‘*balanced*’
Random Forest	64	n_estimators	200, 400
		max_depth	none, ‘10’
		max_features	sqrt, log2
		min_samples_split	2, 5
		min_samples_leaf	1, 2
		class_weight	none, ‘*balanced*’
Gradient Boosting	8	n_estimators	100, 200
		learning_rate	0.05, 0.1
		max_depth	3
		subsample	0.85, 1.0

**Table 3:** Frequency of severe complications observed in 612 stallions after orchiectomy.

Severe Post-Operative Complications Observed	Number of Stallions in Which the Severe Complication Was Observed	Proportion Among Stallions in Which Severe Complications Were Observed
Colic	11	21.2%
Continued stallion-like behaviour	9	17.3%
Evisceration	1	1.9%
Funiculitis	15	28.8%
Haemorrhage	5	9.6%
Scrotal infection	16	30.8%

**Table 4:** Results (median (interquartile range)) of models that produced the best discrimination measures for each of the three Machine Learning tools used for the prediction of development of severe complications after orchiectomies in stallions.

Machine Learning Tool	Hyperparameter	Domain/ Values	Discrimination Measures ^1^					
			[1]	[2]	[3]	[4]	[5]	[6]
Logistic Regression	solver	*liblinear*	0.9130 ^2^ (0.0000)	0.8336 (0.0000)	0.9130 (0.0000)	0.8715 (0.0000)	0.6706 (0.0533)	0.2174 (0.0580)
	penalty	*l*2						
	C	1.0						
	class_weight	none						
Random Forest	n_estimators	400	0.9130 (0.0000)	0.8336 (0.0000)	0.9130 (0.0000)	0.8715 (0.0000)	0.6767 (0.0695)	0.1630 (0.0497)
	max_depth	‘10’						
	max_features	sqrt						
	min_samples_split	5						
	min_samples_leaf	2						
	class_weight	none						
Gradient Boosting	n_estimators	100	0.9076 (0.0109)	0.8336 (0.0335)	0.9076 (0.0109)	0.8714 (0.0116)	0.6645 (0.1019)	0.1740 (0.0619)
	learning_rate	0.05						
	max_depth	3						
	subsample	1.0						
*p* value			<0.0001	0.19	<0.0001	0.033	0.29	<0.0001

^1^ Discrimination measures: [1] accuracy, [2] precision, [3] recall, [4] F1 score, [5] ROC AUC (Receiver operating characteristic Area under the curve), and [6] PR AUC (Precision recall Area under the curve); ^2^ median (interquartile range).

## Data Availability

The data presented in this study are available on request from the corresponding author.

## Supplementary Materials

The following supporting information can be downloaded at: https://www.mdpi.com/article/10.3390/ani16030377/s1, Table S1: Standard pre-operative procedures carried out to all horses prior to orchiectomy; Table S2: Analgesic–anaesthetic procedures performed to horses for orchiectomy; Table S3: Brief description of surgical techniques employed for orchiectomy in horses; Table S4: Brief description of methods employed for haemostasis to horses in cases of orchiectomy; Table S5: Breeds of 612 horses that undertook orchiectomy and were included in a dataset for development of an explainable Machine Learning computational model for the prediction of severe complications after orchiectomy; Table S6: Mean absolute SHapley Additive exPlanations values for the importance of six independent variables employed in the prediction of development of severe complications after orchiectomy in stallions, through the use of three Machine Learning tools; Table S7: Mean absolute SHapley Additive exPlanations values for three surgical techniques employed in the prediction of development of severe complications after orchiectomy in stallions, through the use of three Machine Learning tools; Figure S1: Box-and-whisker plot of accuracy for the model with the best discrimination metrics within each Machine Learning tool employed for the prediction of severe complications after orchiectomy in stallions (blue plot: Logistic Regression, red plot: Random Forest, grey plot: Gradient Boosting); Figure S2: Box-and-whisker plot of F1 score for the model with the best discrimination metrics within each Machine Learning tool employed for the prediction of severe complications after orchiectomy in stallions (blue plot: Logistic Regression, red plot: Random Forest, grey plot: Gradient Boosting); Figure S3: Box-and-whisker plot of age of the stallions, in which post-orchiectomy severe complications were (pink) or were not (green) observed.
