# Supplementary material for: Development of an Explainable Machine Learning Computational Model for the Prediction of Severe Complications After Orchiectomy in Stallions
Source: Animals (Basel). 2026 Jan 25;16(3):377. doi: 10.3390/ani16030377 (PMC12897072; doi:10.3390/ani16030377)
Supplement: Supplementary file 1 [file animals-16-00377-s001.zip › animals-4087329-supplementary.pdf]

Development of an Explainable Machine Learning Computational Model for the Prediction of Severe Complications after Orchiectomy in Stallions

P. Tyrnenopoulou, D. Kalatzis, Y. Kiouvrekis, E. Flouraki, L. Folias, E. Loukopoulos [1], A. Starras, P. Chalvatzis, V. Tsioli, V.S. Mavrogianni, and G.C. Fthenakis [1]

Table S1. Standard pre-operative procedures carried out to all horses prior to orchiectomy.

Time prior to the planned operation	Procedures
Six (6) weeks	confirmation of anti-tetanus vaccination of horses or, if animals had not been previously vaccinated, instigation of a full vaccination regime
One (1) month	restraint from mating activity
One (1) day	detailed general clinical examination and examination of the genital system
Twelve (12) hours	animal fasting
Two (2) hours	refrain from water

Table S2. Analgesic - anaesthetic procedures performed to horses for orchiectomy.

Animal position	Sedation – Pre-medication	Anaesthesia	Local analgesia
Standing position	Intravenous administration of detomidine (20 -40 μg per kg bodyweight (bw)) and butor-phanol (10 μg per kg bw)	---	Aseptic infiltration of 10 mL 2% lidocaine into each testis
Recumbency	Intravenous administration of (a) romifidine (60–80 μg per kg bw) with butorphanol (10 μg per kg bw) or (b) detomidine (20-40 μg per kg bw) with buto-rphanol (10 μg per kg bw)	Intravenous administration of midazolam (0.1 mg per kg bw) and ketamine (2.5 mg per kg)	Aseptic infiltration of 10 mL 2% lidocaine into each testis

Table S3. Brief description of surgical techniques employed for orchiectomy in horses.

Surgical Technique Employed	Description
Open orchiectomy technique	In the open technique, the parietal tunic was incised and left open following removal of the testis. This technique required minimal dissection and could be performed in standing sedated animals or in recumbent animals under anaesthesia. However, because the parietal tunic was opened, there was a potential for communication with the abdominal cavity [Schumacher 2012, Kilcoyne et al. 2013, Rodden et al. 2024].
Semi-closed orchiectomy technique	The semi-closed technique involved initial dissection of the parietal tunic from the scrotal fascia, followed by an incision through the tunic to allow exteriorisation of the testis, epididymis, and a portion of the spermatic vasculature and cord. The spermatic vasculature was then either ligated or emasculated before transection. The parietal tunic could be independently crushed, transected, or suture-closed [Kilcoyne et al. 2013, Kilcoyne and Spier 2021].
Closed orchiectomy technique	In the closed technique, the parietal tunic was dissected free from the scrotal fascia, but was not incised. The intact tunic was removed together with the testis and epididymis. Haemostasis and closure of the tunic were achieved by the crushing action of an emasculator or by applying a ligature [Schumacher 2012, Kilcoyne et al. 2013, Baldwin 2024].

References.

Schumacher, J. Testis. In: *Equine Surgery*, 4th ed.; Auer, J.A.; Stick, J.A., Eds.; Saunders: St. Louis, MO, USA, 2012; pp. 804–840.

Kilcoyne, I.; Watson, J.L.; Kass, P.H.; Spier, S.J. Incidence, management, and outcome of complications of castration in equids: 324 cases (1998–2008). *J. Am. Vet. Med. Assoc.* **2013**, *242*, 820–825.

Baldwin, C.M. A review of prevention and management of castration complications. Equine Vet. Educ. 2024, 36, 97–106.

Kilcoyne, I.; Spier, S.J. Castration complications: a review of castration techniques and how to manage complications. *Vet. Clin. N. Am. Equine Pract.* **2021**, *37*, 259–273.

Rodden, E.B.K.; Suthers, J.M.; Busschers, E.; Burford J.H.; Freeman, S.L. A scoping review on intraoperative and postoperative surgical castration complications in domesticated equids. *Equine Vet. J.* **2024**, *56*, 1115–1128.

Table S4. Brief description of methods employed for haemostasis to horses in cases of orchiectomy.

Procedure applied for haemostasis	Description
Use of the Henderson instrument	The use of the Henderson instrument involved a drill-activated equipment that grasped and twisted the spermatic cord, in order to achieve haemostasis through torsion rather than crushing or ligation. The rapid rotation resulted in controlled tearing and sealing of the vessels, reducing the likelihood of post-operative haemorrhage. Notably, the use of the instrument requires careful alignment of the cord and consistent rotational speed, in order to produce effective vascular occlusion [Schumacher 2012, Kilcoyne et al. 2013, Robert et al. 2017, Racine et al. 2019].
Ligation of the testicular artery	During ligation of the testicular artery, the spermatic cord was isolated and the testicular artery was secured with an absorbable ligature prior to transection of the cord. The ligation provided direct mechanical control of the arterial flow and was often selected in horses, in which it was deemed the potential for increased risk of post-operative haemorrhage, e.g., older animals. After ligation of the artery, the spermatic cord was transected distally to the ligature [Schumacher 2012, Baldwin 2024].
Ligation of the testicular artery with concurrent inguinal ring suturing	Rarely, ligation of the testicular artery was combined with suturing of the inguinal ring. This additional step was performed, in order to reduce the risk of evisceration in horses with a large external inguinal ring or a suspected predisposition to herniation. After vascular ligation, the superficial inguinal ring was partially closed using absorbable sutures to minimize potential communication with the abdominal cavity [Schumacher 2012, Robert et al. 2017].
Use of the Reimer emasculator	The Reimer emasculator provides a two-step mechanism, where crushing and cutting of the spermatic cord occurred separately. The jaws of the emasculator first crushed the cord to occlude the vasculature, and only after complete crush-ing had occurred, the cutting blade was activated to sever the cord. That way, effective haemostasis was achieved by allowing a longer compression phase before the transection. In this method, the correct instrument orientation and the allowance of an adequate time to fully crush the spermatic cord were essential to prevent post-operative bleeding [Schumacher 2012, Comino et al. 2018].
Use of the Reimer emasculator with ligation of the testicular artery	Occasionally, the Reimer emasculator was used in conjunction with ligation of the testicular artery. This procedure enhanced haemostatic control in horses deemed to be at higher risk for excessive post-operative haemorrhage. After ligation of the artery, the emasculator was applied distally to the ligature, with the aim to sever the spermatic cord, while reinforcing vascular occlusion through the application of dual haemostatic mechanisms [Schumacher 2012, Comino et al. 2018].

References.

Schumacher, J. Testis. In: *Equine Surgery*, 4th ed.; Auer, J.A.; Stick, J.A., Eds.; Saunders: St. Louis, MO, USA, 2012; pp. 804–840.

Kilcoyne, I.; Watson, J.L.; Kass, P.H.; Spier, S.J. Incidence, management, and outcome of complications of castration in equids: 324 cases (1998–2008). *J. Am. Vet. Med. Assoc.* **2013**, *242*, 820–825.

Robert, M.P.; Chapuis, R.J.J.; de Fourmestraux, C.; Geffroy, O.J. Complications and risk factors of castration with primary wound closure: retrospective study in 159 horses. *Can. Vet. J.* **2017**, *58*, 466–471.

Racine, J.; Vidondo, B.; Ramseyer, A.; Koch, C. Complications associated with closed castration using the Henderson equine castration instrument in 300 standing equids. *Vet. Surg.* **2019**, *48*, 21–28.

Baldwin, C.M. A review of prevention and management of castration complications. *Equine Vet. Educ.* **2024**, *36*, 97–106.

Comino, F.; Giusto, G.; Caramello, V.; Gandini, M. Do different characteristics of two emasculators make a difference in equine castration? *Equine Vet. J.* **2018**, *50*, 141-144.

Table S5. Breeds of 612 horses that undertook orchiectomy and were included in a dataset for development of an explainable Machine Learning computational model for the prediction of severe complications after orchiectomy.

Horse breed	No. of horses included in the dataset
Arabian	13 (2.1%)
Cob	9 (1.5%)
Haflinger	91 (14.9%)
Lippizaner	1 (0.2%)
Mix breed	188 (30.7%)
Pura Raza Española	6 (1.0%)
Shetland pony	112 (18.3%)
Skyrian	19 (3.1%)
Standardbred	1 (0.2%)
Thoroughbred	80 (13.1%)
Warmblood	92 (15.0%

Figure S1. Box-and-whisker plot of accuracy for the model with the best discrimination metrics within each Machine Learning tool employed for the prediction of severe complications after orchiectomy in stallions (blue plot: Logistic Regression, red plot: Random Forest, grey plot: Gradient Boosting).

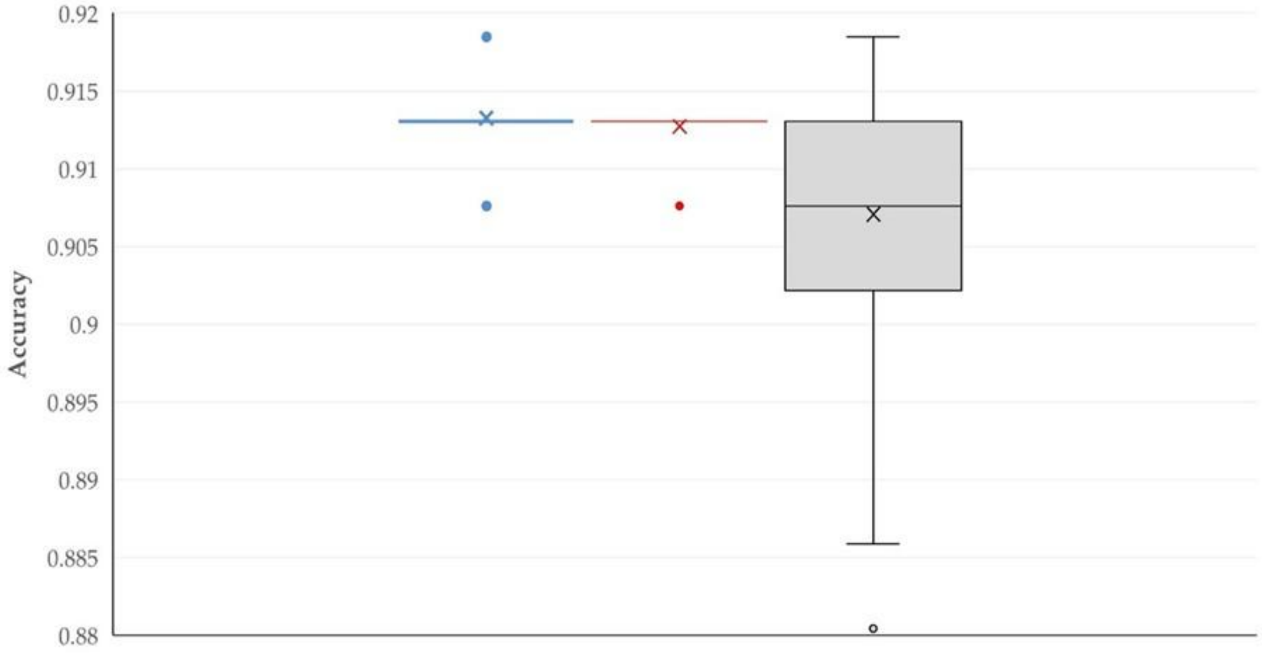

Figure S2. Box-and-whisker plot of F1 score for the model with the best discrimination metrics within each Machine Learning tool employed for the prediction of severe complications after orchiectomy in stallions (blue plot: Logistic Regression, red plot: Random Forest, grey plot: Gradient Boosting).

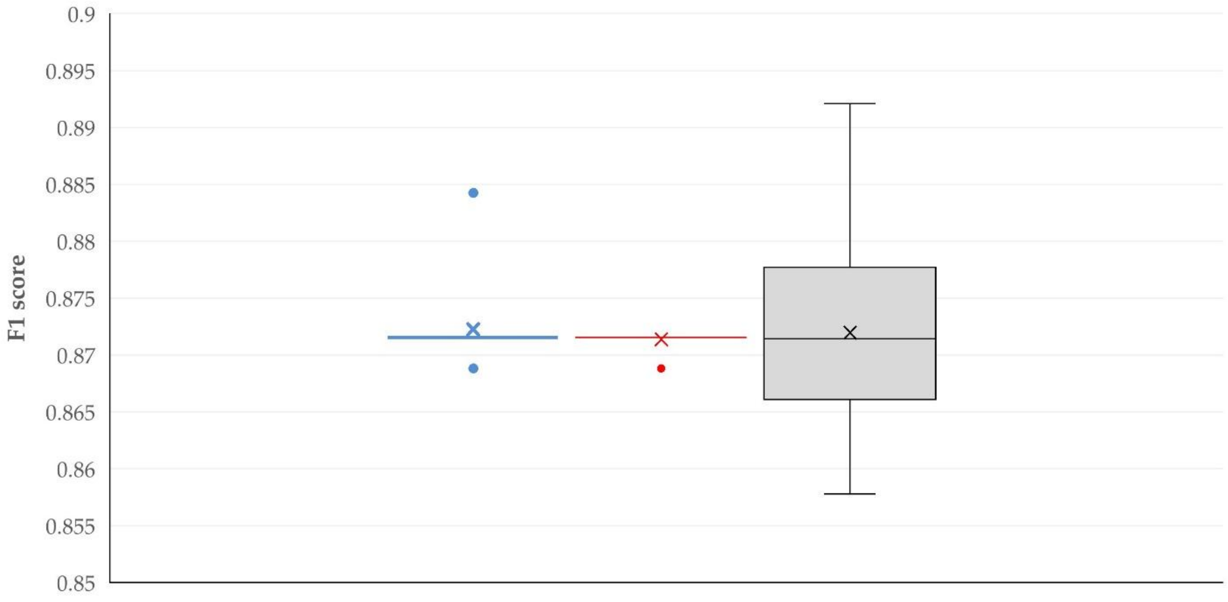

Table S6. Mean absolute SHapley Additive exPlanations values for the importance of six independent variables employed in the prediction of development of severe complications after orchiectomy in stallions, through the use of three Machine Learning tools.

Independent variables	Machine Learning Tools		
	Logistic Regression	Random Forest	Gradient Boosting
Age of horse	0.029823712	0.035167641	0.522772574
Bodyweight of horse	0.008293300	0.021147991	0.295217511
Breed of horse	0.002085054	0.016410151	0.174481548
Haemostasis procedure	0.028568104	0.025848543	0.269780715
Position during operation	0.004421612	0.003617743	0.075042087
Surgical technique	0.033593060	0.027947956	0.400752854

Figure S3. Box-and-whisker plot of age of the stallions, in which post-orchiectomy severe complications were (pink) or were not (green) observed.

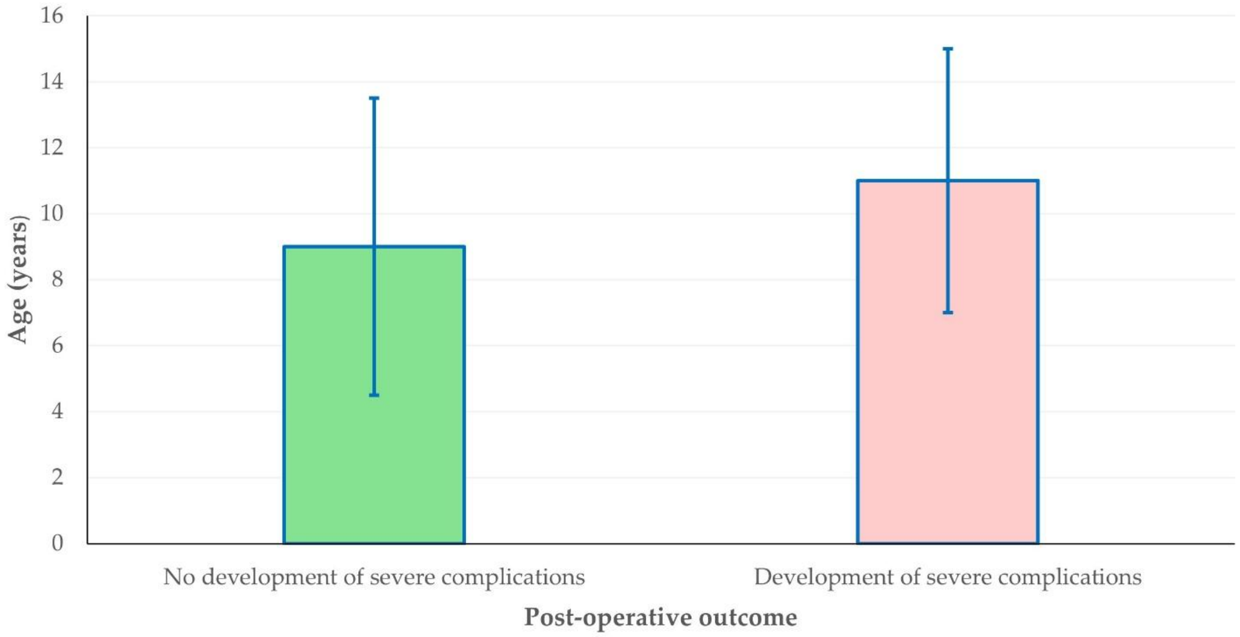

Table S7. Mean absolute SHapley Additive exPlanations values for three surgical techniques employed in the prediction of development of severe complications after orchiectomy in stallions, through the use of three Machine Learning tools.

Categories of the independent variable	Machine Learning Tools		
	Logistic Regression	Random Forest	Gradient Boosting
Open orchiectomy technique	0.012967219	0.016119613	0.688714897
Semi-closed orchiectomy technique	0.003049145	0.003758866	0.182723717
Closed orchiectomy technique	0.007654192	0.008209065	0.218384242
